# Risk Factors of Right Ventricular Dysfunction and Adverse Cardiac Events in Patients with Repaired Tetralogy of Fallot

**DOI:** 10.3390/ijerph181910549

**Published:** 2021-10-08

**Authors:** Benedetta Leonardi, Camilla Calvieri, Marco Alfonso Perrone, Arianna Di Rocco, Adriano Carotti, Massimo Caputo, Aurelio Secinaro, Davide Curione, Maria Giulia Gagliardi, Paolo Guccione, Sonia Albanese, Lorenzo Galletti, Fabrizio Drago

**Affiliations:** 1 Department of Cardiology and Cardiac Surgery, Bambino Gesù Children’s Hospital IRCCS, 00165 Rome, Italy; benedetta.leonardi@opbg.net (B.L.); camilla.calvieri@yahoo.it (C.C.); adriano.carotti@opbg.net (A.C.); mgiulia.gagliardi@opbg.net (M.G.G.); paolo.guccione@opbg.net (P.G.); sonia.albanese@opbg.net (S.A.); lorenzo.galletti@opbg.net (L.G.); fabrizio.drago@opbg.net (F.D.); 2 Department of Public Health and Infectious Diseases, Sapienza University of Rome, 00185 Rome, Italy; Arianna.dirocco@uniroma1.it; 3 Bristol Medical School, University of Bristol, Bristol BS2 8HW, UK; M.Caputo@bristol.ac.uk; 4 Department of Radiology, Bambino Gesù Children’s Hospital IRCCS, 00165 Rome, Italy; aurelio.secinaro@opbg.net (A.S.); davide.curione@opbg.net (D.C.)

**Keywords:** Tetralogy of Fallot, cardiac magnetic resonance imaging, right ventricle dysfunction, repaired Tetralogy of Fallot, pulmonary valve

## Abstract

Aim: This study evaluates the risk factors associated with right ventricular (RV) dilation and dysfunction leading to pulmonary valve replacement (PVR) or adverse cardiac events in repaired Tetralogy of Fallot (rToF) patients. Methods: Data from all rToF patients who underwent magnetic resonance imaging (MRI) evaluation at our hospital between February 2007 and September 2020 were collected. Results: Three hundred and forty-two patients (60% males, 42% older than 18 years), with a median age of 16 years (IQR 13–24) at the time of MRI, were included. All patients underwent complete repair at a median age of 8 months (IQR 5–16), while palliation was performed in 56 patients (16%). One hundred and forty-four patients (42%) subsequently received pulmonary valve replacement (PVR). At the multivariate analysis, male gender was an independent predictor for significant RV dilation, RV and left ventricular (LV) dysfunction. Transventricular ventricular septal defect (VSD) closure and previous palliation significantly affected LV function and RV size, respectively. Male gender and the transventricular VSD closure were independent predictors for PVR. Conclusions: Male gender and surgical history (palliation, VSD closure approach) significantly affected the long-term outcomes in rToF patients and should be taken into consideration in the follow-up management and in PVR timing in this patient population.

## 1. Background

Chronic pulmonary regurgitation (PR) is a common consequence of the surgical repair of Tetralogy of Fallot (rToF) and may result in right ventricular (RV) dilation and dysfunction, decreased exercise tolerance, ventricular arrhythmia, and sudden cardiac death. Pulmonary valve replacement (PVR) can improve functional class, decrease or normalize RV volume, and reduce the risk of arrhythmias [1,2]. However, PVR does not improve RV function once it is already impaired [3,4]. Hence, the procedure should be performed before the development of overt RV failure. The threshold of RV dilatation linked to irreversible RV dysfunction can vary from patient to patient. Furthermore, the evidence on the impact of PVR on RV remodeling, QRS duration and arrhythmia risk is also conflicting [5,6]. On the other hand, the operative risk of PVR, and more importantly, the limited life expectancy of prosthetic valves (especially in younger patients), needs to be taken into consideration. At present, it is still not clear whether the benefits of PVR outweigh the complications associated with the operation and the limited life expectancy of the prosthetic valves currently used. In addition, there are no specific PVR criteria for the pediatric population (<18 years old) or for gender. In asymptomatic rToF patients with significant PR, the proposed criteria for PVR are currently based mainly on RV size measured at MRI [7,8,9], with the potential risk of performing surgery too early. This is probably due to the incomplete understanding of all factors involved in RV dilation and dysfunction and of all predictors of negative long-term outcomes after ToF repair. Therefore, our study aims to investigate the risk factors associated with RV dilation and dysfunction that lead to PVR or adverse cardiac events in a large cohort of rToF children and adolescents.

## 2. Materials and Methods

### 2.1. Patient Population

All patients with rToF, who underwent MRI scans between February 2007 and September 2020 at the Bambino Gesù Children’s Hospital, Rome, were included in the study. Timing for the first MRI assessment was indicated by qualitative echocardiography evaluation showing moderate or severe RV dilation. Patients with the following significant confounding congenital heart defects were excluded: common atrioventricular canal, heterotaxy syndrome, double outlet RV, major aorto-pulmonary collateral arteries, RV-to-pulmonary artery conduits, significant (at least moderate) regurgitation of the aortic and/or the mitral valve. Only rToF patients with transannular or infundibular patches were included. Syndromic rToF patients who were collaborative at MRI were also included. No patient who underwent MRI had prior PVR. Surgical data concerning the timing and type of surgery (Blalock–Taussig shunt followed by repair or primary repair) were reported. The primary repair strategy included: (1) transventricular repair of the VSD with a transannular patch, (2) transatrial repair of the VSD with a transannular patch and (3) transatrial repair of the VSD with an infundibular patch. In addition, echocardiography data (estimate of the RV pressure) and MRI parameters were collected. Finally, we included the last clinical evaluation of each patient with the record of all adverse clinical events. All-cause mortality, aborted cardiac arrest, documented ventricular fibrillation, sustained VT lasting 30 s or longer, atrial arrhythmias (atrial fibrillation, atrial flutter or supraventricular tachycardia), non-sustained VT and/or pacemaker implantation were recorded at follow-up. The documentation of the above-mentioned arrhythmias was obtained by a 24-h Holter monitor or by an ECG during the arrhythmic episode at the hospital. In agreement with the Declaration of Helsinki, all patients enrolled gave written informed consent during their evaluation, stating that their data and images may be subsequently used for research purposes. For participants under 18 years old, a parent and/or the legal tutor gave informed consent. The study was approved by the ethics committee (prot. number: 341/2015). 

### 2.2. MRI Imaging

MRI examinations were performed on a 1.5 T scanner (in our hospital, we used an Achieva scanner, Philips Medical, Best, The Netherlands up to 2014, and afterwards, an Avanto scanner, Siemens, Erlangen, Germany). We followed a study protocol for patients with rToF that included multiple sequences to assess anatomy, cine steady-state free-precession sequences for volume and function assessment, and phase-contrast imaging for flow assessment at the tricuspid, pulmonary and aortic valve, and at both pulmonary arteries [9,10,11]. The slice thickness for short-axis views with full coverage of both ventricles was 5 mm with an interslice gap of 2 mm (Achieva), or 7 mm without a gap (Avanto). 

### 2.3. Image Analysis

The acquired data were analyzed offline on a separate workstation using cardiac post-processing software (Viewforum, Philips Medical, Best, The Netherlands or Mass plus Version 4 and FLOW Version 4, MEDIS, Medical Imaging Systems, Leiden, The Netherlands). Assessment of LV and RV volumes was performed by manual segmentation of the endocardial border of both ventricles on short-axis cine images at the end-diastole and the end-systole, and the volumes were calculated using the disc method [12]. Trabeculations and papillary muscles were considered as part of the ventricular cavity [13]. The ejection fraction (EF) was calculated from the measured volumes. All volumes were indexed by body surface area, calculated using the formula of DuBois et al. (BSA (m^2^) = 0.007184 × Height (cm)^0.725^ × Weight (kg)^0.425^) and compared with the normal values published by Kawel-Boehm et al. [14]. Blood flow was calculated from phase-contrast images using a semiautomatic edge-detection algorithm with operator correction. The regurgitant fraction was calculated as the retrograde flow divided by the forward flow. Pulmonary regurgitation was considered mild if the regurgitant fraction was <20%, moderate between 20 and 40%, and severe >40%. Pulmonary artery/RV outflow tract (RVOT) obstruction was diagnosed when two or more of the following MRI criteria were fulfilled (in addition to RV pressure ≥45 mmHg in echocardiography): (1) flow velocity across the RV outflow tract or a branch pulmonary artery ≥3 m/s; (2) abnormal pulmonary artery size; (3) blood flow maldistribution (RPA < 40%; LPA < 20%). RV dysfunction was considered to be present when RVEF values were ≤47%, whereas LV dysfunction was indicated by RVEF ≤ 55%. PVR was taken into consideration in asymptomatic patients when two or more the following MRI parameters were present: RV end-diastolic volume indexed by BSA (RVEDVi) ≥ 150 mL/m^2^; RV end-systolic volume indexed by BSA (RVESVi) ≥ 80 mL/m^2^; RVEF ≤ 47%; PRF ≥ 40%; LVEF ≤ 55%; moderate or greater tricuspid regurgitation; RV outflow tract obstruction with RV systolic pressure ≥ 0.7 systemic [8]. From 2019 onwards, following the new guidelines for congenital heart disease patients, we have decided to perform PVR for RVEDVi values ≥ 160 mL/m^2^ [7].

### 2.4. Statistical Analysis

All continuous variables were assessed for normality with the Shapiro–Wilk test and by examination of their histogram. Data are presented as frequencies and percentages, mean ± standard deviation, or median and interquartile range, as appropriate. Comparisons between independent groups were performed using the independent samples t-test, Mann–Whitney U test, or chi-square test, as appropriate. Univariate logistic regression analysis was used to identify potential clinical or cardiac MRI independent predictors of RV dilation (RVEDVi > 160 mL/m^2^), RV dysfunction (RVEF < 47%), RVESVi > 80 mL/m^2^, and LV dysfunction (LVEF < 55%). The potential predictors examined were sex, history of palliation, age at repair, repair type, approach for ventricular septal defect (VSD) closure, interval since the repair, presence of RVOT obstruction, and pulmonary regurgitant fraction (PRF). The final multivariate regression model was selected via a stepwise approach involving the minimization of the Akaike Information Criterion. 

Overall survival was defined as the time span from the date of repair until the date of PVR or last follow-up. The Kaplan–Meier estimator was used to estimate survival curves. Univariate and multivariate analyses were performed using the Cox proportional hazard model to assess potential prognostic factors of PVR. Model selection was performed in a stepwise fashion by minimizing the Akaike Information Criterion.

All tests were two-tailed, and analyses were performed using the open-source software R 4.0.4 and related packages.

## 3. Results

### 3.1. Study Cohort Characteristics 

A total of 342 rToF patients underwent MRI examination between February 2007 and September 2020. Their characteristics are reported in Table 1. No patient had undergone a further repair for re-obstruction of the RV outflow tract. Forty-three (13%) were affected by genetic syndromes; in particular, 12 had Down syndrome, 14 had Di George syndrome, and 17 had Catch, Noonan, Goldenhar, Vacterl or Waardenburg syndromes. One hundred and forty-four patients (42%) included in this study underwent subsequent PVR at a mean time of 16.3 + 6 years after the repair. Nine percent of patients experienced adverse cardiac events (Table 1).

### 3.2. MRI Parameters Correlations

RVEDVi was significantly correlated with PRF (rho = 0.661, *p* < 0.0001), RVESVi (rho = 0.874, *p* < 0.0001), RVEF (rho = −0.346, *p* < 0.001) and LVEF (rho = −0.151, *p* = 0.005). Despite similarly increased RV pressure in patients with stenosis of the pulmonary arteries or RV outflow tract obstruction (52 ± 10 vs. 56 ± 13 mmHg, *p* = 0.29), only the latter had significantly smaller RVEDVi (121 ± 24 vs. 140 ± 34 mL/m^2^, *p* = 0.007). In addition, RVEDVi was influenced, albeit weakly, by the time between surgery and MRI (rho = −0.108, *p* = 0.045), but not by the age at repair (*p* = 0.690). RVESVi was correlated with RVEDVi, RVEF, PRF and LVESVi (all *p* < 0.0001). RVEF was negatively linked to age at repair (rho = −0.132, *p* = 0.015) and to time between surgery and MRI (rho = −0.128, *p* = 0.018), in addition to PRF, RVEDVi and the RVEDVi/LVEDVi ratio (all *p* < 0.001). Age at repair and time between surgery and MRI also affected the LVEF (respectively, rho = −0.160, *p* = 0.003, and rho = −0.128, *p* = 0.018). The type of first surgery (palliation followed by repair vs. primary repair) did not influence RV dilation, but patients with BT shunt had lower RVEF and LVEF (respectively, RVEF 50.5% (46.0-54.0) vs. 54.8% (50.0–68.0), *p* < 0.0001, and LVEF 55.0% (51.0–59.0) vs. 57.0% (54.0–62), *p* = 0.010). Finally, the transventricular approach affected RVEF (*p* = 0.001) but not LVEF (*p* = 0.086). 

### 3.3. Post-Hoc Analysis 

#### 3.3.1. Characteristics of Males and Females

MRI parameters according to gender are reported in Table 2. Males presented worse dimensions and function of both ventricles than females (all *p* < 0.001). They also received subsequent PVR more frequently. No difference in adverse cardiac events was reported between the two groups.

#### 3.3.2. Characteristics of Pediatric and Adult Patients

In order to analyze the effect of age on MRI and clinical outcomes, we divided our population into two groups (younger and older than 18 years). Adult patients more frequently had a BT shunt and an infundibular patch, with older age at repair (Table 3); a transannular patch, when performed, was predominantly positioned with a transventricular approach in this group (Table 3). Patients over 18 also had a worse EF of both ventricles, without any significant differences in RV dimensions (Table 3). Moreover, a significantly negative correlation between RVEDVi and age at MRI and the time between surgery and MRI (respectively, rho = −0.184, *p* = 0.02, and rho = −0.198, *p* = 0.018) was highlighted in adults. The prevalence of adverse cardiac events was similar in both groups.

#### 3.3.3. Characteristics of Patients with an RVEDVi ≥ 160 mL/m^2^

Eighty-nine (26%) patients reached RVEDVi 160 mL/m^2^ at MRI evaluation, a median of 15.8 (IQR 12.8–22.1) years after corrective surgery. Among them, 18 (20%) had a RVEF < 47% and 37 (42%) had a LVEF < 55%. Most patients were males, with a mildly higher PRF, a higher prevalence of transannular patch (*p* = 0.001) and a lower prevalence of RVOT obstruction (*p* = 0.009). There were no significant differences concerning the time from corrective surgery, the presence of BT shunt palliation, the age at repair and stenosis of the pulmonary arteries (Table 4). It is clear that patients with RVEDVi ≥ 160 mL/m^2^ had similar characteristics to those who performed PVR after the first MRI. We did not find any difference in adverse cardiac events between the two groups (Table 4).

#### 3.3.4. Characteristics of Patients Who Had a Transannular Patch with a Transventricular Approach

Table 5 summarizes the clinical and MRI characteristics of patients who underwent repair with a ventriculotomy vs. a transatrial-transpulmonary approach. Interestingly, all MRI parameters, except LVEF and PRF, were significantly different between the two groups (Table 5). Patients who had ToF repair with the transventricular approach more frequently reached PVR.

#### 3.3.5. Predictors of RV Dilation and Dysfunction

At multivariate analysis for predictors of RV dilatation >160 mL/m^2^, male gender (OR 2.809, 95% CI 1.540–5.124), transannular patch with the transventricular approach (OR 4.995, 95% CI 2.306–10.820) and time between surgery and MRI (OR 0.886, 95% CI 0.838–0.936) were found to be independent predictors.

At multivariate analysis for predictors of RVESVi > 80 mL/m^2^, male gender (OR 2.672, 95% CI 1.417–5.039), transannular patch with the transventricular approach (OR 5.228, 95% CI 2.353–11.616) and time between surgery and MRI (OR 0.888, 95% CI 0.839–0.941) were found to be independent predictors.

At multivariate analysis for predictors of RV dysfunction (RVEF < 47%), male gender (OR 3.532, 95% CI 1.613–7.736) and BT Shunt (OR 4.539, 95% CI 2.163–9.527) were found to be independent predictors.

#### 3.3.6. Predictors of LV Dysfunction

At multivariate analysis for predictors of LV dysfunction (LVEF < 55%), male gender (OR 1.795, 95% CI 1.092–2.949) and transannular patch with the transventricular approach (OR 2.450, 95% CI 1.484–4.044) were found to be independent predictors.

#### 3.3.7. Time to Event Analysis and Predictors of PVR

**Figure 1:**
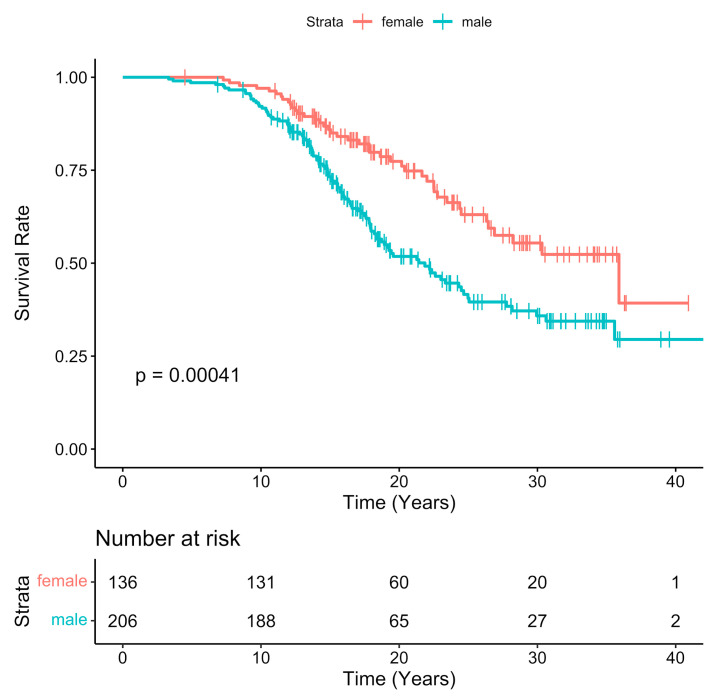
Freedom rate from PVR at the long-term follow-up according to gender.

The median time of the entire cohort was 24.7 years (95% CI: 22.5–30.6). The time to event assessment showed that the median time to PVR event in male patients was 21.9 years compared to 35.9 years for female patients (*p* < 0.001) (Figure 1). Univariate analysis for predictors of PVR identified factors such as male gender (HR 1.894, IC 95% 1.321–2.714), and time between surgery and MRI (HR 0.644, IC 95% 0.603–0.689). At multivariate analysis, male gender and time between surgery and MRI were confirmed as independent variables associated with PVR. All results are shown in Table 6 and Table 7.

## 4. Discussion

In this study, we demonstrated that gender, type of initial operation (pulmonary-systemic shunt vs. corrective surgery), patient age at repair, type of procedure and time interval from corrective surgery, play a significant role in right and left ventricular remodeling in rToF patients. Each factor appears to have a different role in RV response to chronic pulmonary regurgitation, which varies from patient to patient. The combination of these factors ultimately seems to determine the rate of progression of right ventricular dilation. 

Our results reiterate the importance of sex in congenital heart defects [15]. RV growth and function in this population seem to be different among genders, with males being more prone to develop RV dilation and, consequently, to undergo PVR earlier than females [1,16,17], in addition to having a lower RVEF. Indeed, the relationship between gender and PVR indications for rToF patients was recently analyzed in a large multinational study [18], which confirmed that male and female hearts with rToF were distinct, and that the adaptive response of cardiac dimensions and systolic function to chronic volume overload appeared to vary according to age and sex.

We found a negative correlation between RV dilation and the time between surgery and MRI. This finding, in contrast with the study by Lee et al. [1,19], which identified a longer interval since repair as a risk factor for RV dilation, could be explained by the theory suggested by Greutmann et al. [20]. In fact, the author hypothesized that RV remodeling occurs early after initial surgical repair and is followed by a “steady state” characterized by a dilated but stable RV. However, a considerably higher number of rToF patients should be followed with serial MRI over many years in order to confirm this hypothesis. Alternatively, the different MRI characteristics emerging from the comparison between pediatric and adult populations, as well as the negative correlation between RVEDVi and the time from surgery, could be the expression of a different RV responses to chronic relevant pulmonary insufficiency from patient to patient. In fact, as suggested by Frigiola et al., there could be a subgroup of rToF patients in whom RV volume does not reach a significant dilation due to a relatively more benign course of the disease and to an ideal anatomic substrate and optimal surgical therapy [21]. Furthermore, as already shown by Spiewak et al. [22], RV outflow tract obstruction limits RV dilation. Surprisingly enough, this does not seem to happen with pulmonary branch stenosis. Our results emphasize that the development of RV dysfunction [23] is also affected either by age at surgical repair [24,25] or by progressive RV dilation secondary to pulmonary regurgitation, as reported by other studies [24,25,26]. Indeed, a late primary surgical correction may lead to long-term RV and/or LV dysfunction due to preoperative hypoxemia, as suggested by Hausdorf et al. [27] and confirmed by our data. 

In addition to the late primary surgical repair, the transventricular approach for VSD closure seems to be an independent predictor for significant RV dilation (RVEDVi ≥ 160 mL/m^2^, RVESVi ≥ 80 mL/m^2^), LV dysfunction, and PVR in both univariate and multivariate analysis. Nevertheless, there is still no agreement in the literature on which technique (transventricular vs. transatrial-transpulmonary approach) can give the best results in the long-term for rToF patients [28,29,30,31,32]. However, there is no doubt that ventriculotomy can lead to unfavorable side effects such as transmural scarring and increased wall stress and strain [33], which, in turn, can contribute to a long-term impairment of RV function and, consequently, LV function. 

Finally, in our cross-sectional study, the adverse clinical events occurred late during follow-up after rToF and were quite uncommon. Moreover, these adverse events did not appear to be affected by gender, age of the patient, significant RV dilation ≥ 160 mL/m^2^, and/or use of the transventricular approach. This could be explained by the young age and the preserved ejection fraction of most patients of our cohort, in accordance with what was reported by other studies [25,34], which identified significantly depressed LVEF and RVEF as predictors of major adverse outcomes and reported that the prevalence of sustained tachyarrhythmias increases with age [35].

## 5. Limitations

This is a single-center cross-sectional retrospective study that could potentially have been affected by selection bias. As a matter of fact, patients were scheduled for MRI only when moderate to severe RV dilation was qualitatively detected by echocardiography. Therefore, this clinical approach may have caused a selection bias of patients with a more severe dilation. Our study did not take into consideration rToF patients who had a right ventricle-to-pulmonary artery conduit, a history of pulmonary atresia or an absent pulmonary valve corrected with a transannular or infundibular patch. Furthermore, we did not include the cardiopulmonary exercise stress test data; therefore, we cannot draw conclusions about the impact of MRI parameters and severe RV dilation on exercise performance.

## 6. Conclusions

Adverse cardiac events at follow-up after repair of ToF are low and uncommon in young adults with preserved cardiac systolic function. Male gender and surgical strategy are independent risk factors for increased RV dilatation and/or dysfunction, LV dysfunction and PVR in rToF patients. Therefore, they should probably become part of the decision-making process for the timing of PVR in asymptomatic patients, in addition to the usual MRI parameters already included in the PVR indication criteria.

## Figures and Tables

**Table 1:** Patients Characteristics. Legend: BT shunt = Blalock–Taussig shunt; TP = transannular patch; BSA = body surface area; BMI = body mass index; RVEDVi = right ventricular end-diastolic volume indexed by BSA; RVESVi = right ventricular end-systolic volume indexed by BSA; RVEF = right ventricular ejection fraction; LVEDVi = left ventricular end-diastolic volume indexed by BSA; LVESVi = left ventricular end-systolic volume indexed by BSA; LVEF = left ventricular ejection fraction; PR = pulmonary regurgitation fraction; RVOT = right ventricular outflow tract; PAs = pulmonary arteries; PVR = pulmonary valve replacement; NS-VT = non-sustained ventricular arrhythmias; S-VT = sustained ventricular arrhythmias; AA = Atrial arrhythmia; PMK = pacemaker.

Male sex, n (%)	206 (60.2)
BT shunt, n (%)	56 (16.4)
Age at shunt (months) (median, IQR)	4 (1–9)
Age at repair (months) (median, IQR)	8 (5–16)
Transannular Patch, n (%)	317 (92.7)
Transventricular approach, n (%)	168 (49.1)
Age at cardio MRI (yrs) (median, IQR)	15.8 (12.5–24.3)
Patients = >18 years old at cardio MRI	142 (41.5)
Time between surgery and cardiac MRI (yrs) (median) IQR)	15 (12–21)
BSA (median, IQR)	1.58 (1.36–1.77)
BMI (median, IQR)	20.8 (18.4–24.1)
RVEDVi mL/m^2^ (median, IQR)	135.4 (113.7–160.7)
RVEDVi ≥ 160, n (%)	89 (26.0)
RVESVi mL/m^2^ (median, IQR)	93.50 (71.0–122.0)
RVESVi ≥ 80, n (%)	78 (22.8)
RVEF < 47%, n (%)	46 (13.4)
RVEF, % (median, IQR)	50.0 (49.0–58.0)
LVEDVi mL/m^2^ (median, IQR)	79.95 (70.2–89.0)
LVESVi mL/m^2^ (median, IQR)	33.50 (27.9–40.0)
LVEF, % (median, IQR)	57.0 (53.0–61.3)
LVEF < 55%, n (%)	107 (31.3)
PR,% (median, IQR)	41.0 (32.2–50.9)
RVOT and/or PAs branches stenosis, n (%)	28 (13.0)
PVR, n (%)	144 (42.1)
Adverse cardiac events:	
- NS-VT	19 (5.5)
- AA	8 (2.4)
- S-VT	1 (0.3)
- PMK	2 (0.6)
- Sudden death	1 (0.3)
- Death for other causes	1 (0.3)

**Table 2:** Clinical and MRI characteristics according to gender. Legend: BT shunt = Blalock–Taussig shunt; TP = transannular patch; BSA = body surface area; BMI = body mass index; RVEDVi = right ventricular end-diastolic volume indexed by BSA; RVESVi = right ventricular end-systolic volume indexed by BSA; RVEF = right ventricular ejection fraction; LVEDVi = left ventricular end-diastolic volume indexed by BSA; LVESVi = left ventricular end-systolic volume indexed by BSA; LVEF = left ventricular ejection fraction; PR = pulmonary regurgitation fraction; RVOT = right ventricular outflow tract; PAs = pulmonary arteries; PVR = pulmonary valve replacement; NS-VT = non-sustained ventricular arrhythmias; S-VT = sustained ventricular arrhythmias; AA = Atrial arrhythmia; PMK = pacemaker.

	Male (n = 206)	Female (n = 136)	*p* Value
Age at repair, months, median (IQR)	8.0 (5.0–16.0)	9.0 (5.0–18.0)	0.180
Age at BT shunt, days, median (IQR)	139.0 (39.0–191.0)	82.0 (9.0–311.5)	0.754
BT shunt, n (%)	25 (12.0)	31 (22.8)	0.014
Transannular Patch, n (%)	190 (92.2)	127 (93.4)	0.851
Transventricular approach, n (%)	105 (51.0)	63 (46.3)	0.465
Age at cardio MRI, years, median (IQR)	15.6 (12.4–22.4)	16.6 (12.9–25.6)	0.151
Time between surgery and cardiac MRI, years, median (IQR)	14.7 (11.5–21.0)	15.4 (11.8-24.2)	0.158
BSA, median (IQR)	1.7 (1.4-1.9)	1.5 (1.4-1.6)	<0.001
BMI, median (IQR)	21.0 (18.4–24.0)	20.7 (18.5–24.2)	0.782
RVEDVi mL, median (IQR)	225.7 (180.9–277.0)	187.0 (150.5–218.5)	<0.001
RVEDVi ≥ 160, n (%)	70 (34.0)	19 (14.0)	<0.001
RVEDVi mL/m^2^, median (IQR)	144.7 (123.7–169.7)	128.6 (104.0–147.2)	<0.001
RVESVi mL, median (IQR)	103.5 (79.2–130.7)	82.0 (63.2–101.9)	<0.001
RVESVi ≥ 80 mL/m^2^, n (%)	61 (29.6)	17 (12.5)	<0.001
RVESVi mL/m^2^, median (IQR)	66.4 (53.2–83.4)	56.2 (44.0–70.8)	<0.001
RVEF%, median (IQR)	53.0 (48.1–56.6)	56.0 (51.0–59.2)	<0.001
RVEF < 47%, n (%)	36 (17.5)	10 (7.3)	0.012
LVEDVi mL/m^2^, median (IQR)	83.2 (75.2–93.9)	72.7 (67.0–82.5)	<0.001
LVESVi mL/m^2^, median (IQR)	35.8 (30.3–43.0)	30.1 (25.1–34.9)	<0.001
LVEF%, median (IQR)	56.0 (52.0–60.0)	58.1 (55.0–63.0)	0.001
LVEF < 55%, n (%)	75 (36.4)	32 (23.5)	0.017
PR%, median (IQR)	42.7 (33.0–51.7)	40.0 (32.0–50.0)	0.570
RVOT and/or PAs branches stenosis, n (%)	28 (13.6)	16 (11.7)	0.742
PVR, n (%)	102 (49.5)	42 (30.9)	<0.001
Adverse cardiac events:			
- NS-VT, n (%)	8 (3.9)	11 (8.1)	0.548
- AA, n (%)	2 (1.0)	6 (4.4)	
- S-VT, n (%)	1 (0.5)	0 (0.0)	
- PMK, n (%)	0 (0.6)	2 (1.5)	
- Sudden death, n (%)	0 (0.0)	1 (0.7)	
- Death for other causes, n (%)	0 (0.0)	1 (0.7)	

**Table 3:** Clinical and MRI characteristics according to age. Legend: BT shunt = Blalock–Taussig shunt; TP = transannular patch; BSA = body surface area; BMI = body mass index; RVEDVi = right ventricular end-diastolic volume indexed by BSA; RVESVi = right ventricular end-systolic volume indexed by BSA; RVEF = right ventricular ejection fraction; LVEDVi = left ventricular end-diastolic volume indexed by BSA; LVESVi = left ventricular end-systolic volume indexed by BSA; LVEF = left ventricular ejection fraction; PR = pulmonary regurgitation fraction; RVOT = right ventricular outflow tract; PAs = pulmonary arteries; PVR = pulmonary valve replacement; NS-VT = non-sustained ventricular arrhythmias; S-VT = sustained ventricular arrhythmias; AA = atrial arrhythmia; PMK = pacemaker.

	Age < 18 (n = 200)	Age = >18 (n = 142)	*p* Value
Males, n (%)	126 (63.0)	80 (56.3)	0.259
Age at repair, months, median (IQR)	6 (4.0–10.0)	16 (7.0–27.7)	<0.001
Age at BT shunt, days, median (IQR)	36.0 (16.0–89.5)	139.0 (38.0–286.5)	0.055
BT shunt, n (%)	11 (5.5)	45 (31.7)	<0.001
Transannular Patch, n (%)	183 (91.5)	134 (94.4)	0.428
Transventricular approach, n (%)	33 (16.5)	135 (67.5)	<0.001
Age at cardio MRI, years, median (IQR)	12.9 (10.9–15.1)	25.7 (21.9–31.3)	<0.001
Time between surgery and cardiac MRI, median (IQR)	12.3 (10.2–14.4)	24.4 (20.1–29.4)	<0.001
BSA, median (IQR)	1.45 (1.19–1.65)	1.71 (1.59–1.93)	<0.001
BMI, median (IQR)	19.9 (17.5–22.7)	22.6 (20.4–25.4)	<0.001
RVEDVi mL/m^2^, median (IQR)	139.8 (115.9–160.8)	131.7 (105.9–160.8)	0.465
RVEDVi ≥ 160, n (%)	51 (26.0)	38 (27.0)	0.891
RVESVi mL/m^2^, median (IQR)	62.8 (46.9–77.7)	62.1 (50.1–79.5)	0.754
RVESVi ≥ 80, n (%)	43 (21)	35 (25)	0.580
RVEF %, median (IQR)	56.0 (50.0–59.0)	53.0 (49.0–55.3)	<0.001
RVEF < 47%, n (%)	22 (11)	24 (17)	0.157
LVEDVi mL/m^2^, median (IQR)	78.9 (70.2–88.0)	81.9 (70.3–92.4)	0.116
LVESVi mL/m^2^, median (IQR)	32.8 (27.2–38.2)	34.2 (29.1–42.4)	0.042
LVEF % (median, IQR)	58.0 (54.4–62.0)	56.0 (52.0–59.6)	0.006
LVEF < 55%, n (%)	53 (27.0)	54 (38.0)	0.032
PR%, median (IQR)	42 (33.8–52.0)	40 (29.2–50.0)	0.103
RVOT and/or PAs branches stenosis, n (%)	27 (13.5)	17 (12)	0.677
PVR, n (%)	92 (46.0)	52 (36.0)	0.105
Adverse cardiac events:			
- NS-VT, n (%)	8 (4.0)	11 (7.7)	0.186
- AA, n (%)	3 (1.5)	5 (3.5)	
- S-VT, n (%)	0 (0.0)	1 (0.7)	
- PMK, n (%)	2 (1.0)	0 (0.0)	
- Sudden death, n (%)	0 (0.0)	1 (0.7)	
- Death for other causes, n (%)	1 (0.5)	1 (0.0)	

**Table 4:** Clinical and MRI characteristics according to RVEDVi ≥ 160 mL/m^2^. Legend: BT shunt = Blalock–Taussig shunt; TP = transannular patch; BSA = body surface area; BMI = body mass index; RVEDVi = right ventricular end-diastolic volume indexed by BSA; RVESVi = right ventricular end-systolic volume indexed by BSA; RVEF = right ventricular ejection fraction; LVEDVi = left ventricular end-diastolic volume indexed by BSA; LVESVi = left ventricular end-systolic volume indexed by BSA; LVEF = left ventricular ejection fraction; PR = pulmonary regurgitation fraction; RVOT = right ventricular outflow tract; PAs = pulmonary arteries; PVR = pulmonary valve replacement; NS-VT = non-sustained ventricular arrhythmias; S-VT = sustained ventricular arrhythmias; AA = atrial arrhythmia; PMK = pacemaker.

	RVEDVi < 160 (n = 253)	RVEDVi ≥ 160 (n = 89)	*p* Value
Males, n (%)	136 (53.8)	70 (78.7)	<0.001
Age at repair, months, median (IQR)	8.0 (5.0–16.0)	11 (5.0–17.0)	0.386
Age at BT shunt, days, median (IQR)	138.5 (46.2–269.5)	36.0 (5.0–186.0)	0.124
BT shunt, n (%)	38 (15.0)	18 (46.1)	0.330
Transannular patch, n (%)	232 (91.7)	85 (95.5)	0.342
Transventricular, n (%)	114 (45.0)	54 (60.7)	0.016
Age at cardio MRI, years, median (IQR)	15.83 (13.4–25.6)	15.8 (12.8–22.1)	0.305
Time between surgery and MRI, median (IQR)	15.21 (11.7–24.2)	14.6 (11.4–19.3)	0.132
BSA, median (IQR)	1.6 (1.4–1.8)	1.5 (1.2–1.8)	0.219
BMI, median (IQR)	21.2 (18.9–24.7)	19.7 (17.6–22.1)	<0.001
RVEDVi mL/m^2^, median (IQR)	127.1 (106.3–142.5)	176.8 (168.9–193.0)	<0.001
RVESVi mL/m^2^, median (IQR)	56.5 (44.8–66.3)	86.6 (77.7–98.8)	<0.001
RVESVi ≥ 80 mL/m^2^, n(%)	17 (6.7)	61 (68.5)	<0.001
RVEF%, median (IQR)	54.0 (50.0–59-0)	51.0 (48.0–56.0)	<0.001
RVEF < 47%, n (%)	28 (11.1)	18 (20.2)	0.049
LVEDVi mL/m^2^, median (IQR)	76.8 (68.6–84.0)	89.0 (82.8–98.2)	<0.001
LVESVi mL/m^2^, median (IQR)	31.3 (26.0–36.9)	40.0 (34.0–45.1)	<0.001
LVEF%, median (IQR)	57.0 (51.0–61.0)	57.0 (54.0–62.0)	0.149
LVEF < 55%, n (%)	70 (27.7)	37 (41.6)	0.021
PR%, median (IQR)	38.0 (28.0–47.0)	52.0 (44.0–56.0)	<0.01
RVOT and/or PAs branches stenosis, n (%)	36 (14.2)	8 (9.0)	0.277
PVR, n (%)	67 (26.5)	77 (85.5)	<0.001
Adverse cardiac events:			
- NS-VT, n (%)	13 (5.1)	6 (6.7)	0.111
- AA (%)	7 (2.8)	1 (1.1)	
- S-VT, n (%)	1 (0.4)	0 (0.0)	
- PMK, n (%)	0 (0.0)	2 (2.2)	
- Sudden death, n (%)	0 (0.0)	1 (1.1)	
- Death for other causes, n (%)	1 (0.4)	0 (0.0)	

**Table 5:** Clinical and MRI characteristics according to VSD closure approach. Legend: BT shunt = Blalock–Taussig shunt; TP = transannular patch; BSA = body surface area; BMI = body mass index; RVEDVi = right ventricular end-diastolic volume indexed by BSA; RVESVi = right ventricular end-systolic volume indexed by BSA; RVEF = right ventricular ejection fraction; LVEDVi = left ventricular end-diastolic volume indexed by BSA; LVESVi = left ventricular end-systolic volume indexed by BSA; LVEF = left ventricular ejection fraction; PR = pulmonary regurgitation fraction; RVOT = right ventricular outflow tract; PAs = pulmonary arteries; PVR = pulmonary valve replacement; NS-VT = non-sustained ventricular arrhythmias; S-VT = sustained ventricular arrhythmias; AA = atrial arrhythmia; PMK = pacemaker.

	Transventricular (n = 168)	Transatrial (n = 174)	*p* Value
Males, n (%)	105 (62.5)	101 (58.0)	0.465
Age at repair, months, median (IQR)	14.0 (7.0–25.0)	6.0 (4.0–10.0)	<0.001
Age at BT shunt, days, median (IQR)	132.5 (35.7–273.2)	36.0 (16.0–89.5)	0.161
BT shunt, n (%)	48 (28.6)	8 (4.6)	<0.001
Transannular Patch, n (%)	160 (9.5)	157 (90.2)	0.116
Age at cardio MRI, years, median (IQR)	24.6 (19.0–30.6)	12.6 (10.7–14.5)	<0.001
Time between surgery and MRI, median (IQR)	22.7 (17.8–28.1)	11.8 (10.1–13.8)	<0.001
BSA, median (IQR)	1.7 (1.6–1.9)	1.4 (1.2–1.6)	<0.001
BMI, median (IQR)	22.0 (20.0–25.0)	19.8 (17.0–22.9)	<0.001
RVEDVi mL/m^2^ (median, IQR)	142.6 (118.4–169.2)	131 (111.7–151.0)	0.004
RVEDVi ≥160 mL/m^2^, n (%)	54 (32.1)	35 (20.1)	0.016
RVESVi mL/m^2^, median (IQR)	65.1 (53.9–83.5)	59.2 (44.2–72.6)	<0.001
RVESVi ≥80 mL/m^2^, n (%)	49 (29.2)	29 (16.7)	<0.001
RVEF%, median (IQR)	53.0 (49.0–56–0)	56.0 (50.0–59.5)	<0.001
RVEF < 47%, n (%)	30 (59.5)	16 (9.2)	0.029
LVEDVi mL/m^2^, median (IQR)	82.4 (71.3–93.5)	77.9 (69.4–85.0)	0.001
LVESVi mL/m^2^, median (IQR)	35 (30.0–43.3)	31.3 (25.9–37.2)	<0.001
LVEF%, median (IQR)	56.0 (52.0–60.0)	58.0 (55.0–62.0)	0.149
LVEF < 55%, n (%)	67 (39.9)	40 (23.0)	0.001
PR%, median (IQR)	43.2 (33.7–52.0)	39.9 (31.2–50.0)	0.931
RVOT and/or PAs branches stenosis, n (%)	7 (4.2)	18 (10.3)	0.047
PVR, n (%)	82 (48.8)	62 (35.6)	0.018
Adverse cardiac events:			
- NS-VT, n (%)	5 (3.0)	14 (8.0)	0.057
- AA, n (%)	2 (1.2)	6 (3.4)	
- S-VT, n (%)	0 (0.0)	1 (0.6)	
- PMK, n (%)	2 (1.2)	0 (0.0)	
- Sudden death, n (%)	0 (0.0)	1 (0.6)	
- Death for other causes, n (%)	1 (0.6)	0 (0.0)	

**Table 6:** Predictors of RV dilation and dysfunction, LV dysfunction. Legend: RVOT = right ventricular outflow tract; PAs = pulmonary arteries; BT shunt = Blalock–Taussig shunt; TP = transannular patch; TA = transatrial approach; TVA = transventricular approach; IP = infundibular patch; BMI = body mass index; PR = pulmonary regurgitation fraction; Ref = reference.

**RVEDVi > 160 mL/m^2^**								
	**Univariate**				**Multivariate**			
**Variable**	**OR**	**Low IC 95%**	**Up IC 95%**	***p* Value**	**OR**	**Low IC 95%**	**Up IC 95%**	***p* Value**
RVOT and/or PAs branches stenosis, n (%)	0.595	0.266	1.335	0.208				
Gender (male)	3.17	1.803	5.571	<0.001	2.809	1.540	5.124	0.001
BT shunt, n (%)	1.434	0.77	2.671	0.255	2.118	0.998	4.495	0.051
Age at repair	1.01	0.999	1.022	0.068				
TP -with TA -with TVA IP	Ref 1.693 0.68	Ref 1.022 0.222	Ref 2.804 2.143	0.041 0.520	Ref 4.995 0.890	Ref 2.306 0.271	Ref 10.820 2.923	<0.001 0.848
Time between surgery and MRI	0.967	0.935	1.000	0.051	0.886	0.838	0.936	<0.001
BMI	0.898	0.844	0.956	0.001				
PR%	1.097	1.068	1.127	<0.001				
**RVESVi > 80 mL/m^2^**								
	**Univariate**				**Multivariable**			
**Variable**	**OR**	**Low IC 95%**	**Up IC 95%**	***p* Value**	**OR**	**Low IC 95%**	**Up IC 95%**	***p* Value**
RVOT and/or PAs branches stenosis, n (%)	0.605	0.258	1.416	0.247				
Gender (male)	2.945	1.633	5.311	<0.001	2.672	1.417	5.039	0.002
BT shunt, n (%)	1.784	0.951	3.346	0.071	2.568	1.198	5.508	0.015
Age at repair	1.015	1.003	1.026	0.014				
TP -with TA -with TVA IP	Ref 1.916 0.628	Ref 2.245 0.176	Ref 3.261 2.245	0.017 0.474	Ref 5.228 0.811	Ref 2.353 0.217	Ref 11.616 3.035	<0.001 0.756
Time between surgery and MRI	0.975	0.942	1.009	0.150	0.888	0.839	0.941	<0.001
BMI	0.902	0.845	0.962	0.002				
PR%	1.090	1.061	1.119	<0.001				
**RVEF < 47%**								
	**Univariate**				**Multivariate**			
**Variable**	**OR**	**Low IC 95%**	**Up IC 95%**	***p* Value**	**OR**	**Low IC 95%**	**Up IC 95%**	***p* Value**
RVOT and/or PAs branches stenosis, n (%)	0.434	0.129	1.464	0.178				
Gender (male)	2.668	1.276	5.579	0.009	3.532	1.613	7.736	0.002
BT shunt, n (%)	3.413	1.708	6.82	0.001	4.539	2.163	9.527	<0.001
Age at repair	1.013	1.001	1.025	0.037				
TP -with TA -with TVA IP	Ref 2.261 1.393	Ref 1.145 0.370	Ref 4.465 5.242	0.019 0.624				
Time between surgery and MRI	1.012	0.973	1.052	0.557				
BMI	1.026	0.958	1.098	0.461				
PR%	1.025	1.003	1.048	0.028				
**LVEF < 55%**								
	**Univariate**				**Multivariate**			
**Variable**	**OR**	**Low IC 95%**	**Up IC 95%**	***p* Value**	**OR**	**Low IC 95%**	**Up IC 95%**	***p* Value**
RVOT and/or PAs branches stenosis, n (%)	0.91	0.455	1.818	0.79				
Gender (male)	1.861	1.143	3.029	0.013	1.795	1.092	2.949	0.021
BT shunt, n (%)	1.676	0.929	3.024	0.086				
Age at repair	1.002	0.99	1.013	0.77				
TP -with TA -with TVA IP	Ref 2.505 2.505	Ref 1.523 1.031	Ref 4.119 6.085	<0.001 0.043	Ref 2.450 2.438	Ref 1.484 0.996	Ref 4.044 5.967	<0.001 0.051
Time between surgery and MRI	1.016	0.987	1.046	0.278				
BMI	0.942	0.892	0.996	0.034				
PR%	1.022	1.006	1.038	0.006				

**Table 7:** Predictors for PVR. Legend: BSA = body surface area; PR = pulmonary regurgitation fraction.

PVR Events								
	Univariate				Multivariate			
Variable	HR	Low IC 95%	Up IC 95%	*p* Value	HR	Low IC 95%	Up IC 95%	*p* Value
Gender (male)	1.894	1.321	2.714	0.001	1.894	1.317	2.724	0.001
BSA	0.065	0.033	0126	<0.001				
PR%	1.087	0.620	1.909	0.770				
Time between surgery and MRI	0.644	0.603	0.689	<0.001	0.645	0.604	0.689	<0.001

## Data Availability

Not applicable.

## Abbreviations

BT	Blalock–Taussig
TP	transannular patch
MRI	magnetic resonance imaging
BSA	body surface area
BMI	body mass index
RV	right ventricle
LV	left ventricle
RVEDVi	right ventricular end-diastolic volume indexed by BSA
RVESVi	right ventricular end-systolic volume indexed by BSA
RVEF	right ventricular ejection fraction
LVEDVi	left ventricular end-diastolic volume indexed by BSA
LVESVi	left ventricular end-systolic volume indexed by BSA
LVEF	left ventricular ejection fraction
PRF	pulmonary regurgitation fraction
RVOT	right ventricular outflow tract
PAs	pulmonary arteries
PVR	pulmonary valve replacement
NS-VT	non-sustained ventricular arrhythmias
S-VT	sustained ventricular arrhythmias
